# Supplementary material for: Aggregates Associated with Instability of Antibodies during Aerosolization Induce Adverse Immunological Effects
Source: Pharmaceutics. 2022 Mar 18;14(3):671. doi: 10.3390/pharmaceutics14030671 (PMC8949695; doi:10.3390/pharmaceutics14030671)
Supplement: Supplementary file 1 [file pharmaceutics-14-00671-s001.zip › pharmaceutics-1627215-supplementary.pdf]

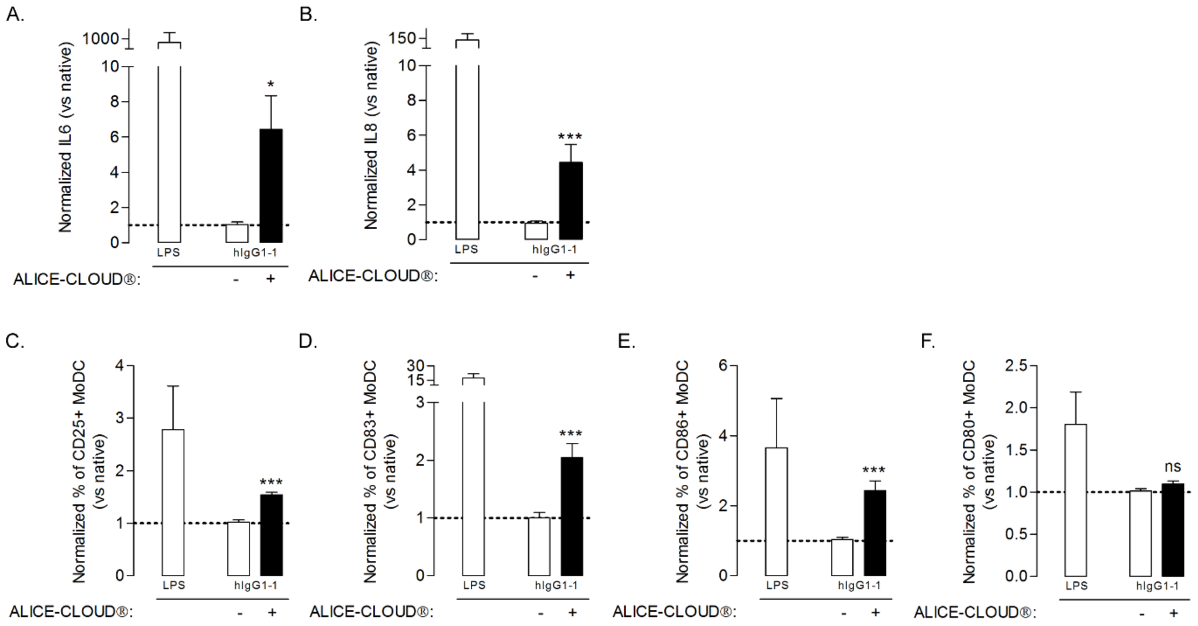

<u>Supplementary Figure S1:</u> MoDC were stimulated using equal final concentration of hIgG1-1 at 100 µg/mL either native (white bars) or nebulized (black bars) using the VITROCELL Cloud 12 system. IL6 (A) and IL8 (B) were quantified in cell-free supernatant after 18h. CD25 (E), CD83 (F), CD86 (G), CD80 (H) expression was measured using flow cytometry after 18h. The data are quoted as the mean ± SEM. *, *** : p<0.05 and p<0.001 respectively, in a t-test. The results are representative of three independent experiments (n=3-6/experiment).

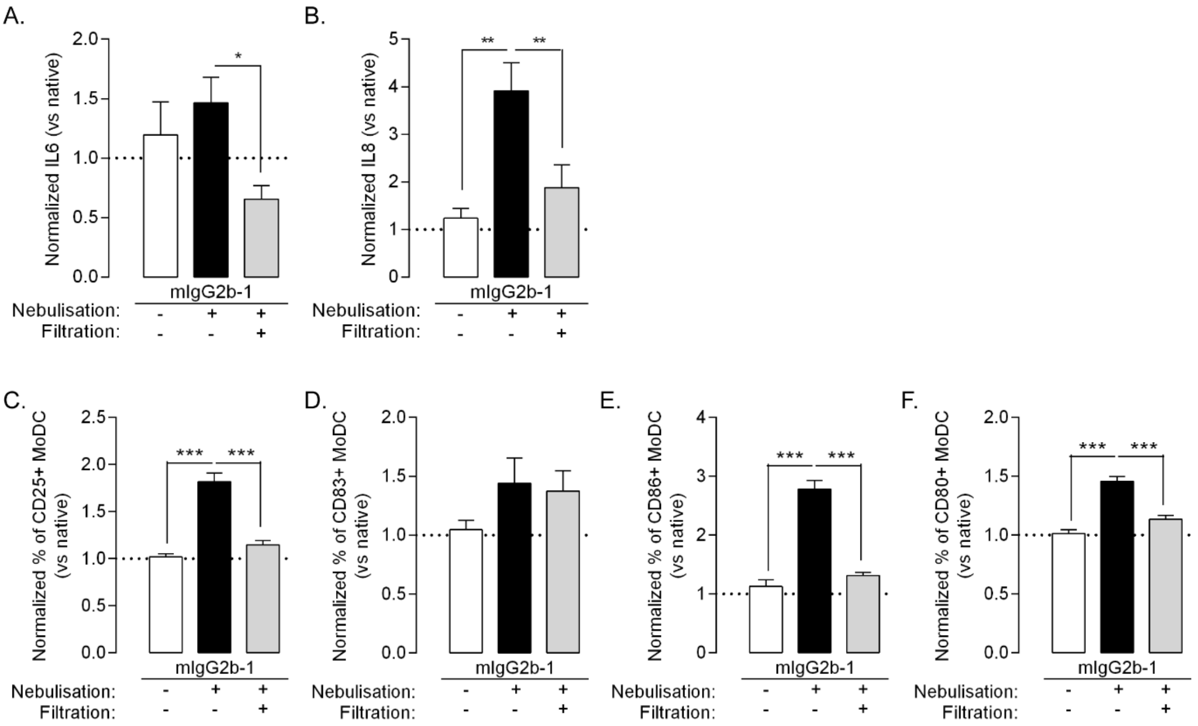

<u>Supplementary Figure S2:</u> mIgG2b-1 and mIgG2b-2 were either nebulized and subsequently collected using an Aeroneb Pro^{TM} vibrating-mesh nebulizer (black bars) and further 0.45µm-filtered (gray bars) or left untreated (white bars). (MoDC were stimulated using 100 µg/mL of mIgG2b-1 for 18 hours. IL6 (A)

and IL8 (B) were quantified in cell-free supernatant. CD25 (C), CD83 (D), CD86 (E), CD80 (F) expression were measured using flow cytometry. The data are quoted as the mean ± SEM. *, **, *** : $p<0.05$, $p<0.01$ and $p<0.001$ respectively, in a one-way ANOVA with Newman-Keuls's correction for multiple comparisons. The results are representative of three independent experiments (n=3-6/experiment).

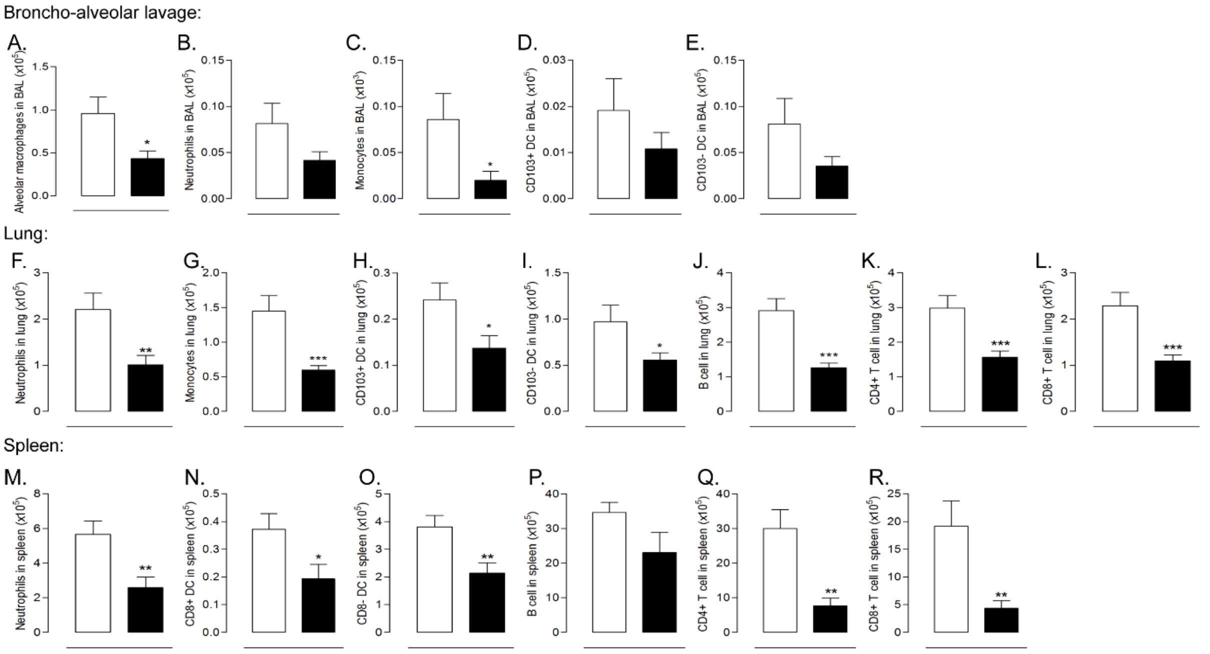

<u>Supplementary Figure S3:</u> C57/BL6jRj mice received a 40 µL orotracheal instillation of mIgG2b-1 at 100 µg/mL either native (white bars) or nebulized (black bars). The total number of alveolar macrophages (A), neutrophils (B, F and M), monocytes (C and G), CD103+ dendritic cells (D and H), CD103- dendritic cells (E and I), B cells (J and P), CD8+ dendritic cells (N), CD8- dendritic cells (O), CD4+ T cells (K and Q) and CD8+ T cells (L and R) were quantified in BAL (A to E), in the lungs (F to L) and the spleen (M to R) using flow cytometry, 18h after the administration. The data are quoted as the mean ± SEM. *, *** : $p<0.05$ and $p<0.001$ respectively, in a one-way ANOVA with *t*-test. The results are representative of three experiment (n=5/experiments).

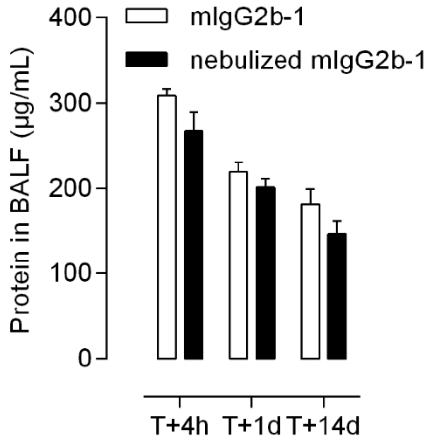

Supplementary Figure S4: C57/BL6jRj mice received a 40 µL orotracheal instillation of mIgG2b-1 at 100 µg/mL either native (white bars) or nebulized (black bars). The concentration of protein in BALF was determined 4 hours, 18 hours and 14 days after the administration. The data are quoted as the mean ± SEM. The results are representative of one experiment (n=5) for 4 hours and 14 days, and representative of three experiments (n=5/experiments) for 18 hours.

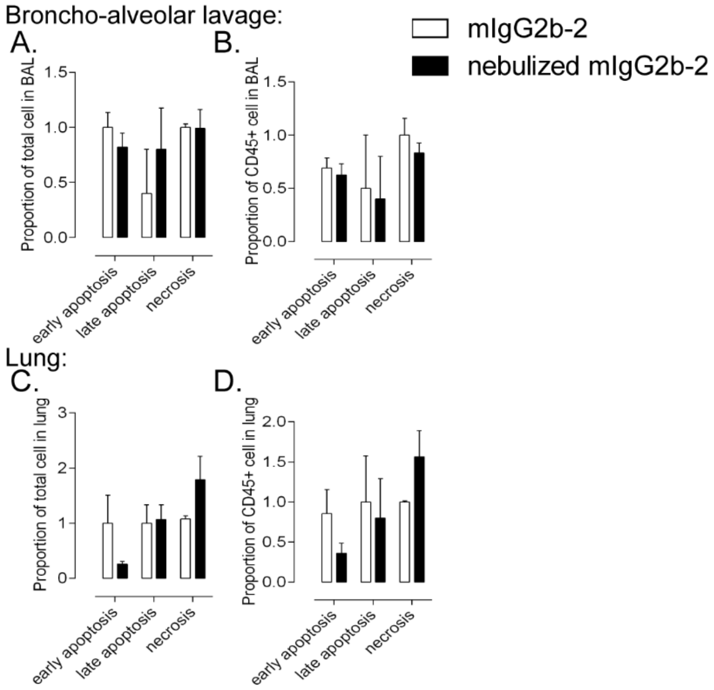

Supplementary Figure S5: C57/BL6jRj mice received a 40 µL orotracheal instillation of mIgG2b2-2 at 100 µg/mL either native (white bars) or nebulized (black bars). The proportion of early apoptotic cells (Annexin-V+/PI-), late apoptotic cells (Annexin-V+/PI+) and necrotic cells (Annexin-V-/PI+) were quantified in total cell (A and C) or CD45+ cell (B and D), 18h after the administration in BAL (A and B) and lungs (C and D) using flow cytometry. The data are quoted as the mean ± SEM. The results are from two experiments (n=5/experiments).

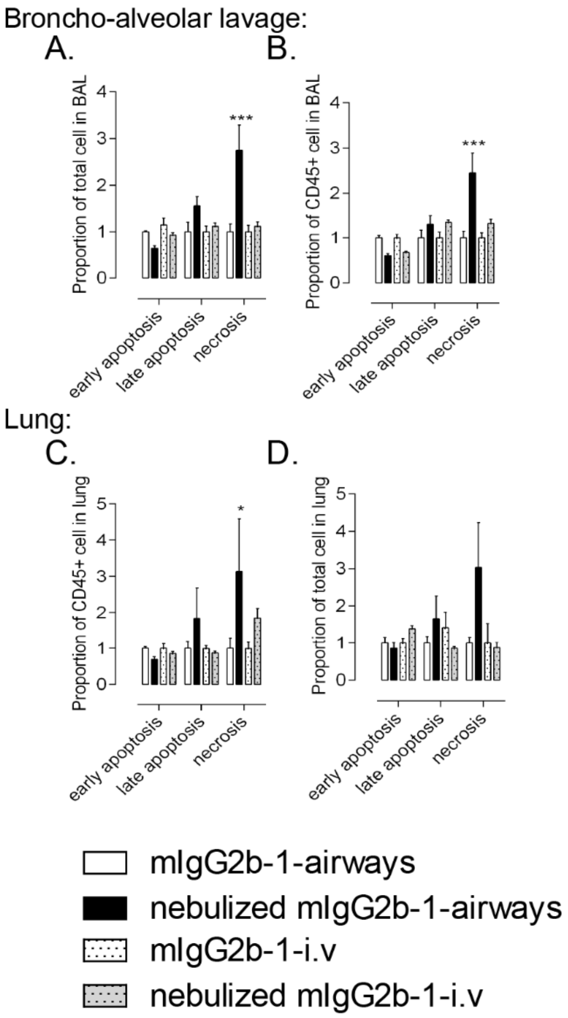

Supplementary Figure S6: C57/BL6jRj mice received a 40 µL orotracheal instillation of mIg2b-1 at 100 µg/mL either native (white bars) or nebulized (black bars) through the airways or through intraveinous injection at D+0, D+7, D+14, D+21 and D+28. The total number of cells (A and C) and CD45+ cells (B and D) were quantified in the BAL (A and B) and in the lungs (C and D) using flow cytometry, 18h after the last administration. The data are quoted as the mean ± SEM. *, ** : p<0.05 and p<0.01 respectively, in a one-way ANOVA with *t*-test. The results are representative of three experiment (n=5/experiments).

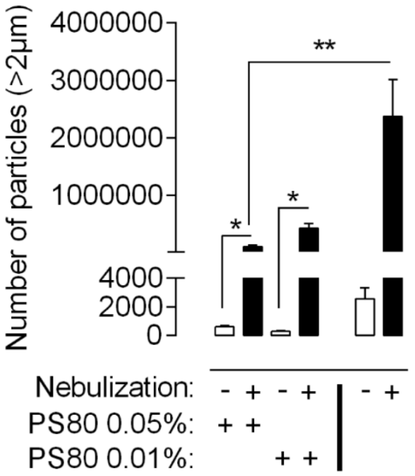

Supplementary Figure S7: mIgG2b-1 formulated in 1X-PBS, 1X-PBS+Polysorbate80 0.01% (PS80 0.01%) or 1X-PBS+Polysorbate80 0.05% (PS80 0.0.5%) was nebulized using an Aeroneb Pro^{TM} vibrating-mesh nebulizer (black bars) or left untreated (white bars) and aerosols were collected. The total number of particles (which size >2µm) was quantified using a flow microscope. The data are quoted as the mean ± SEM. *, **: *p*<0.05 and *p*<0.01 respectively, in a *t*-test. The results are representative of three independent nebulizations (n=3/experiment).

Lung:

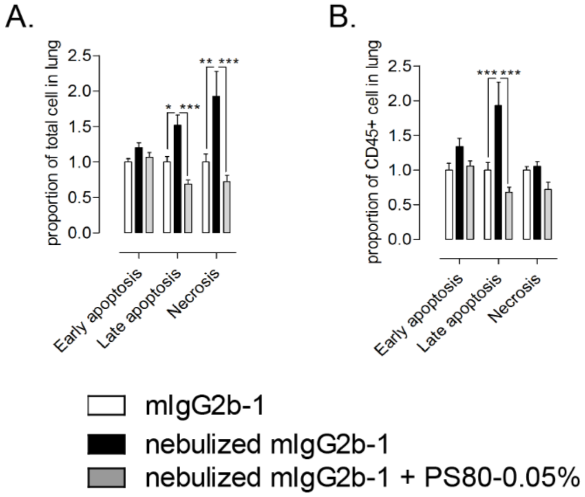

Supplementary Figure S8: C57/BL6jRj mice received a 40 µL orotracheal instillation of mIgG2b-1 at 100 µg/mL either native (white bars), nebulized (black bars) or nebulized + Polysorbate80 0.05% (PS80 0.05%, gray bars). The proportion of early apoptotic cells (Annexin-V+/PI-), late apoptotic cells (Annexin-V+/PI+) and necrotic cells (Annexin-V-/PI+) were quantified in total cell (A) or CD45+ cell (B) 18h after the administration in lungs using flow cytometry. The data are quoted as the mean ± SEM. *, **, *** : *p*<0.05, p<0.01 and *p*<0.001 respectively, in a one-way ANOVA with *t*-test. The results are representative of three experiment (n=5/experiments).

Supplementary Table s1: Dynamic light scattering analysis of antibodies

Antibody	Radius (nm)	%mass	PD index
native hIgG1-1	5,07	99,67	0,18
nebulized hIgG1-1	5,25	99,75	0,02
native hIgG1-2	5,40	99,90	0,12
nebulized hIgG1-2	5,43	99,78	0,16
native hIgG1-3	5,38	99,87	0,05
nebulized hIgG1-3	5,23	99,90	0,17
native mIgG2b-1	5,88	99,80	n/a
nebulized mIgG2b-1	n/a		
native mIgG2b-2	6,30	99,40	0,37
nebulized mIgG2b-2	7,36	93,06	n/a

Supplementary table s2: List of antibodies used for flow cytometry

murine

Target	Fluorophore	Clone	Supplier
CD45	APC Cy7	30-F11	Biolegend
Ly6C	PeCy7	HK1.4	ebiosciences
SiglecF	PE	E50-2440	BD
SiglecF	V450	E50-2440	BD
F4/80	Violet Blue	BM8	Biolegend
Ly6G	FITC	1A8	Biolegend
CD40	APC	2/23	Biolegend
CD11b	PerCP-Cy5.5	M1/70	ebiosciences
CD11b	Pe-Cy7	M1/70	Biolegend
CD11b	Pe-Cy7	M1/70	Biolegend
CD86	V450	GL1	Biolegend
CD80	FITC	16-10A1	Biolegend
CD11c	PE	N418	ebiosciences
CD11c	APC	N418	Biolegend
IA/IE	PercP-EF710	NIMR-4	ebiosciences
IA/IE	PE	NIMR-4	ebiosciences
CD4	APC Cy7	GK1.5	Biolegend
CD4	Pe-Cy7	GK1.5	Biolegend
CD3	PerCP.Cy5.5	17A2	Biolegend
CD8	APC	53-6.7	ebiosciences
CD8	PerCP-Cy5.5	17A2	BD
CD8	Vioblue	53-6.7	ebiosciences
CD62L	FITC	MEL-14	ebiosciences
CD19	APC	1D3	BD
B220/CD45R	PerCP.Cy5.5	RA3-6B2	Biolegend

human

Target	Fluorophore	Clone	Supplier
CD45	APC Cy7	2D1	BD
CD25	APC	BC96	Biolegend
CD80	PE	2D10	Biolegend
CD86	PerCP-Cy5.5	BU63	Biolegend
CD83	FITC	HB15e	Biolegend

Supplementary table S3: phenotype of analyzed cells

Name	Phenotype	Origin
Alveolar macrophages	CD45+ CD11c+ SiglecF+ CD11b- F4/80+	BAL
Neutrophils	CD45+ CD11c- SiglecF- CD11b+ Ly6G+ Ly6C- F4/80-	BAL, Lung, Spleen
Monocytes	CD45+ CD11c- SiglecF+ CD11b+ Ly6G- Ly6C+ F4/80-	BAL, Lung, Spleen
CD103+ Dendritic cells (CD103+ DC)	CD45+ CD11c+ SiglecF- CD11b- CD103+ F4/80- IA/IE+	BAL, Lung
CD103- Dendritic cells (CD103- DC)	CD45+ CD11c+ SiglecF- CD11b+ CD103- F4/80- IA/IE+	BAL, Lung
B cell	CD45+ CD3- CD19+	Lung, Spleen
CD4+ T cell	CD45+ CD19- CD3+ CD4+ CD8-	Lung, Spleen
CD8+ T cell	CD45+ CD19- CD3+ CD4- CD8+	Lung, Spleen
CD8a+ Dendritic cells (CD8a+ DC)	CD45+ CD8a+ CD11c+ CD11b- MHCII+	Spleen
CD8a- Dendritic cells (CD8a+ DC)	CD45+ CD8a- CD11c+ CD11b- MHCII+	Spleen
Early apoptosis	Annexin-V+ PI-	BAL, Lung, Spleen
Late apoptosis	Annexin-V+ PI+	BAL, Lung, Spleen
Necrosis	Annexin-V- PI+	BAL, Lung, Spleen

Supplementary Table S4: Particle size distribution (%) of nebulized antibodies

Antibody	2-5µm	5-25µm	>25µm
native mIgG2b-1	62,1	37,1	0,8
nebulized mIgG2b-1	71,6	27,6	0,8
nebulized mIgG2b-1 + PS80-0,05%	70,7	29,1	0,3
nebulized mIgG2b-1 + 0.45µm	69,2	30,8	0,0

Supplementary Table S5: Dynamic light scattering analysis of nebulized antibodies

Antibody	Radius (nm)	%mass	PD index
native mIgG2b-1	6,00	99,80	18,10
nebulized mIgG2b-1	n/a	n/a	n/a
nebulized mIgG2b-1 + PS80-0,05%	n/a	n/a	n/a
nebulized mIgG2b-1 + 0.45µm	n/a	n/a	n/a

Supplementary Table S6: Nanodrop analysis of antibody concentration

	[IgG] (mg/mL)
native mIgG2b-1	2,8
nebulized mIgG2b-1	2,53
nebulized mIgG2b-1 + PS80-0,05%	2,28
nebulized mIgG2b-1 + 0.45µm	2,79
